# Single-cell analysis reveals essential lncRNAs regulating human trophoblast lineage differentiation

**DOI:** 10.1093/lifemedi/lnag010

**Published:** 2026-04-17

**Authors:** Weiyi Zhang, Chunyan Le, Shanru Yi, Wenqiang Liu, Xiaoyu Liu, Zhen Yin, Hong Wang, Rui Gao, Shaorong Gao, Jiayu Chen

**Affiliations:** Clinical and Translation Research Center of Shanghai First Maternity & Infant Hospital, Shanghai Key Laboratory of Signaling and Disease Research, Frontier Science Center for Stem Cell Research, School of Life Sciences and Technology, Tongji University, Shanghai 200092, China; Clinical and Translation Research Center of Shanghai First Maternity & Infant Hospital, Shanghai Key Laboratory of Signaling and Disease Research, Frontier Science Center for Stem Cell Research, School of Life Sciences and Technology, Tongji University, Shanghai 200092, China; Clinical and Translation Research Center of Shanghai First Maternity & Infant Hospital, Shanghai Key Laboratory of Signaling and Disease Research, Frontier Science Center for Stem Cell Research, School of Life Sciences and Technology, Tongji University, Shanghai 200092, China; Clinical and Translation Research Center of Shanghai First Maternity & Infant Hospital, Shanghai Key Laboratory of Signaling and Disease Research, Frontier Science Center for Stem Cell Research, School of Life Sciences and Technology, Tongji University, Shanghai 200092, China; Clinical and Translation Research Center of Shanghai First Maternity & Infant Hospital, Shanghai Key Laboratory of Signaling and Disease Research, Frontier Science Center for Stem Cell Research, School of Life Sciences and Technology, Tongji University, Shanghai 200092, China; Department of Control Science and Engineering, State Key Laboratory of Autonomous Intelligent Unmanned Systems, Tongji University, Shanghai 201210, China; Shanghai Innovation Institute, Shanghai 200030, China; Clinical and Translation Research Center of Shanghai First Maternity & Infant Hospital, Shanghai Key Laboratory of Signaling and Disease Research, Frontier Science Center for Stem Cell Research, School of Life Sciences and Technology, Tongji University, Shanghai 200092, China; Clinical and Translation Research Center of Shanghai First Maternity & Infant Hospital, Shanghai Key Laboratory of Signaling and Disease Research, Frontier Science Center for Stem Cell Research, School of Life Sciences and Technology, Tongji University, Shanghai 200092, China; Clinical and Translation Research Center of Shanghai First Maternity & Infant Hospital, Shanghai Key Laboratory of Signaling and Disease Research, Frontier Science Center for Stem Cell Research, School of Life Sciences and Technology, Tongji University, Shanghai 200092, China; Clinical and Translation Research Center of Shanghai First Maternity & Infant Hospital, Shanghai Key Laboratory of Signaling and Disease Research, Frontier Science Center for Stem Cell Research, School of Life Sciences and Technology, Tongji University, Shanghai 200092, China

**Keywords:** placenta, lncRNA, protein coding gene, *cis*-regulation, trophoblasts

## Abstract

The human placenta sustains pregnancy through intricate trophoblast lineage dynamics that are critical for fetal development and pregnancy success. While studies on protein-coding genes (PCGs) have advanced our understanding of placental biology, the regulatory roles of noncoding RNAs, particularly long noncoding RNAs (lncRNAs), in trophoblast lineage specification and function remain poorly understood. Here, we profile single-cell lncRNA dynamics across human placental development, revealing distinct cell-type- and gestational stage-specific expression profiles. Integrated analysis revealed that lncRNAs modulate histone modification levels at the regulatory regions of target genes via *cis*-action, thereby regulating the expression of key genes essential for trophoblast differentiation. Functional studies by using *in vivo* and *in vitro* models fully identify *ECEL1P2*–*ALPP, SEMA3B–AS1*–*SEMA3B*, and *MYCNUT*/*MYCNOS*–*MYCN* as pivotal regulatory axes driving cytotrophoblast self-renewal, syncytiotrophoblast fusion, and epithelial–mesenchymal transition, respectively, which are essential for trophoblast identity and function. Notably, dysregulation of lncRNA–PCG pairs in pathological pregnancies underscores the clinical relevance of these noncoding networks. Together, our findings uncover an unappreciated layer of lineage-specific noncoding regulation, providing mechanistic insight and potential biomarkers for placental development and associated disorders.

## Introduction

With the continuous advancement of genomics, it has become clear that, beyond traditional protein-coding genes (PCGs), a vast number of noncoding transcripts play crucial roles in gene regulatory networks. Among these, long noncoding RNAs (lncRNAs) not only outnumber PCGs but also contribute to essential biological functions, including chromosomal stability, cell differentiation, and disease pathogenesis [1]. Compared with mRNAs, lncRNAs exhibit distinct features in transcriptional regulation and processing: their promoters typically recruit fewer transcription factors and epigenetic marks, resulting in generally lower expression levels but stronger cell type specificity [2–5]. Functionally, lncRNAs can fold into specific secondary or tertiary structures that allow them to interact with proteins, participate in chromatin remodeling and transcriptional regulation. Moreover, they can act as *cis*- or *trans*-regulatory elements to modulate gene expression over short or long genomic distances [6–8].

At the genomic level, lncRNAs are frequently positioned near or embedded within PCGs, forming diverse spatial configurations. According to the classification proposed by Luo et al. [9], lncRNA–PCG pairs can be categorized into six configurations: antisense head-to-head (XH), antisense tail-to-tail (XT), intronic antisense (XI), exonic overlapping (XO), sense upstream (SU), and sense downstream (SD). These arrangements suggest that physical proximity may shape transcriptional regulation. Indeed, deletion of lncRNA loci often alters the expression of nearby genes, changes that may be attributed to their embedded DNA regulatory elements or the transcriptional process itself [10–13]. However, whether lncRNAs exert genuine *cis*-regulatory effects through their RNA transcripts remains unresolved and requires further validation. Beyond these mechanistic insights, accumulating evidence from diverse developmental systems highlights the indispensable role of lncRNAs in cell fate decisions. For instance, lncRNAs have been shown to regulate zygotic genome activation (ZGA) during early embryogenesis, to control hematopoietic stem cell emergence, and modulate germ cell differentiation [14–16]. These findings emphasize that lncRNAs are not merely transcriptional noises but critical regulators of development processes.

The human placenta is a transient yet indispensable organ that sustains pregnancy by mediating nutrient and oxygen exchange, endocrine signaling, and immune tolerance at the maternal–fetal interface [17, 18]. Originating from the trophectoderm after implantation [19], placental trophoblasts include cytotrophoblasts (CTBs) that function as progenitors, syncytiotrophoblasts (STBs) that facilitate maternal–fetal exchange, and extravillous trophoblasts (EVTs) that remodel uterine spiral arteries to ensure adequate blood flow [20–22]. The differentiation of CTBs into STBs and EVTs is essential for placental development and maternal–fetal communication [23]. Dysregulation of trophoblast development can result in severe pregnancy complications, including preeclampsia (PE), characterized by defective trophoblast invasion and vascular remodeling [24], and gestational diabetes mellitus (GDM), where maternal hyperglycemia disrupts placental homeostasis [25].

Recent technological advances have significantly improved our understanding of placental biology. The establishment of human trophoblast stem cell (hTSC) lines provides a robust platform for investigating trophoblast proliferation and differentiation [19]. In addition, trophoblast organoids (TOs) allow for physiologically relevant modeling of self-organization and early developmental processes, including embryo implantation [26]. At the molecular level, single-cell transcriptomic analyses have mapped trophoblast heterogeneity and lineage trajectories, revealing key PCG regulatory networks that govern cell fate specification [27]. Although individual lncRNAs, such as *H19*, *MEG3*, and *TUG1*, have been implicated in regulating trophoblast proliferation, invasion, and syncytialization [28–30], the global landscape and functional significance of lncRNAs during early human trophoblast fate decisions remain largely unexplored.

In this study, we systematically analyzed lncRNA expression patterns across human placental development, utilizing single-cell transcriptomic data spanning from the first trimester to term. This resource enables the systematic identification of functionally relevant lncRNAs associated with trophoblast differentiation and maturation. Through molecular validation, we uncovered essential roles for *ECEL1P2*, *SEMA3B–AS1*, *MYCNUT*, and *MYCNOS* in driving trophoblast stemness maintenance, syncytialization, and invasion, mediated by their ability to act in *cis* to remodel histone signatures at the regulatory regions of target genes. Collectively, our study reveals an essential layer of lncRNA-mediated regulation in trophoblast lineage specification, offering new insights into human placental development and its associated disorders.

## Results

### lncRNA expression patterns distinguished by trophoblast subtype and gestational stage

**Figure 1.**
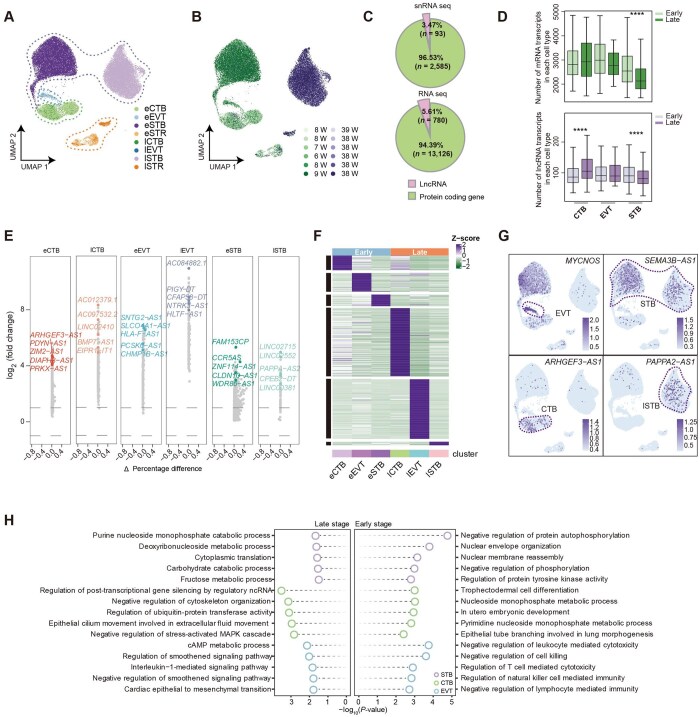
Transcriptomic landscape of lncRNAs in human placental trophoblasts. (A) UMAP plot showing first-trimester and term placental single-nucleus transcriptomes (CTB, cytotrophoblast; EVT, extravillous trophoblast; STB, syncytiotrophoblast; STR, stromal cell; e, early; l, late). (B) UMAP plot showing the distribution of first-trimester and term placental samples. (C) Pie plot of the fraction of lncRNAs and mRNAs in snRNA-seq and bulk RNA-seq, respectively. The value “*n*” indicates the average number of detected transcripts per cell for both lncRNAs and mRNAs based on the experimentally measured data. (D) Box plots of transcript counts in per cell type. Statistical significance was determined by two-sided Wilcoxon rank-sum tests (****P < 0.0001). (E) Volcano plot of cell-type-specific lncRNAs across trophoblast subtypes. The *x*-axis represents the difference in the percentage of cells expressing each gene between the two compared cell types (Δ percentage = pct.1 − pct.2), while the *y*-axis indicates the log_2_(fold change) in average gene expression. This representation integrates both expression magnitude and expression prevalence at the single-cell level. (F) Heatmap of cell-type-specific lncRNA expression atlas across trophoblast populations. (G) UMAP plots showing the expression distribution of representative lncRNAs (Color gradient represents the average expression level per cell). (H) GO biological process terms enriched in protein-coding genes (PCGs) associated with stage-specific lncRNAs.

To comprehensively investigate the roles of lncRNAs in human placental trophoblast self-renewal and lineage differentiation, we analyzed single-nucleus RNA sequencing (snRNA-seq) data from six first-trimester (6–9 weeks) and six term (38–39 weeks) placental samples sourced from publicly available datasets [27] (Figs. 1A, 1B and S1A–D). Given that lncRNAs are frequently enriched in the nucleus [31], the use of snRNA-seq enables robust detection of nuclear-retained and nascent lncRNA transcripts, making it particularly suitable for systematic lncRNA profiling. Transcriptomic analysis revealed that lncRNAs constituted 3.47% of all the transcripts, a proportion that remained remarkably consistent across major trophoblast subtypes and different gestational stages (Figs. 1C and S1E). This proportional stability was further corroborated by a re-analysis of published bulk RNA-seq datasets (Fig. 1C) [19], underscoring the consistent and biologically relevant representation of lncRNAs throughout trophoblast development.

Quantification of transcript counts revealed that the number of lncRNAs was much lower than that of mRNAs. While mRNAs exhibited more pronounced fluctuations across cell types and developmental stages (Fig. 1D), the overall expression trends of lncRNAs paralleled those of mRNAs during trophoblast maturation and differentiation. To further dissect the dynamics underlying these expression patterns, we employed RNA velocity analysis. This revealed that the initiation of differentiation, particularly in early-gestation EVTs, was characterized by high kinetic activity to support invasive and migration plasticity [32]. In contrast, the endpoints of trophoblast differentiation displayed a unified trend: a concurrent decline in both transcript abundance and velocity length (Figs. 1D and S1F). This synchronization reflects a global entry into transcriptional quiescence, marking the transition from high transcriptional activity to a stable functional state.

Furthermore, we identified numerous lncRNAs whose expression was restricted to specific trophoblast subtypes and gestational stages (Fig. 1E). To explore the expression characteristics of lncRNAs during human placental development, we classified lncRNAs into six clusters and delineated their dynamic expression landscape in human trophoblasts (Figs. 1F and S1G). Notably, late-gestation CTBs and EVTs were characterized by a particularly abundant set of cell-type-specific lncRNAs. UMAP visualization further highlighted the differential enrichment of key lncRNAs across trophoblast subtypes (e.g. *MYCNOS*, *SEMA3B–AS1*, *ARHGEF3–AS1*, and *PAPPA2–AS1*) (Fig. 1G). Importantly, the expression patterns of several PCGs closely resembled those of their neighboring lncRNAs, which displayed lineage-specific expression (Figs. 1F and S1H). Gene ontology analysis of PCGs neighboring lncRNAs strongly suggested that they were involved in stage and cell-type-specific regulatory functions, including epithelial morphogenesis regulation in CTBs, immune response in EVTs, and cell fusion and cell metabolism in STBs (Fig. 1H). These results support a model in which lncRNAs may act as *cis*-regulators and highlight that lncRNAs are closely coordinated with the regulatory networks underlying human trophoblast differentiation.

### lncRNAs regulate neighboring PCGs involved in human trophoblast development

**Figure 2.**
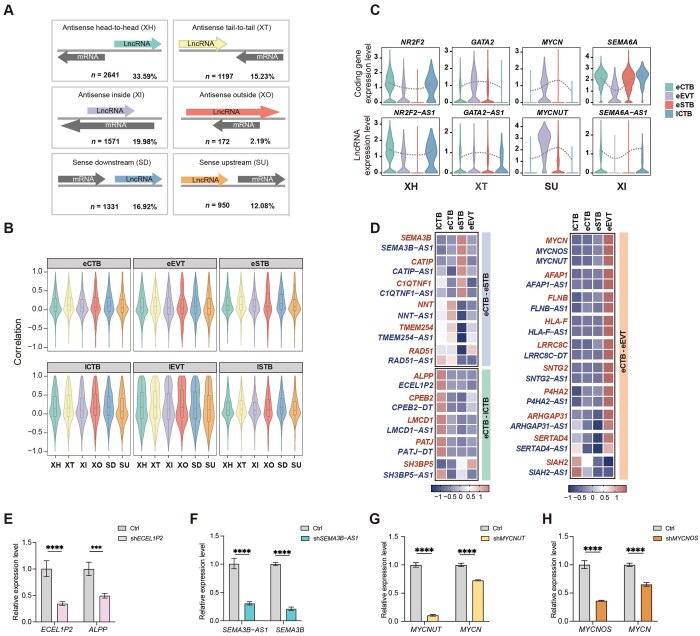
Genomic configuration and co-expression of lncRNA–PCG pairs. (A) Classification of lncRNA–PCG genomic configurations. (B) Expression correlation of lncRNAs with adjacent protein-coding genes across categories. (C) Representative loci showing expression of lncRNAs and adjacent genes across cell types. (D) High-confidence lncRNA–mRNA regulatory pairs across developmental transitions. (E) qRT-PCR analysis of *ECEL1P2* and *ALPP* in hTSCs (human trophoblast cells, *n* = 3 technical replicates). Data were represented as the mean ± SD. Data were analyzed by two-way ANOVA test (****P < 0.0001; ***P < 0.001). (F) qRT-PCR analysis of *SEMA3B–AS1* and *SEMA3B* in Day 6 STB (*n* = 3 technical replicates). Data were represented as the mean ± SD. Data were analyzed by two-way ANOVA test (****P < 0.0001). (G, H) qRT-PCR analysis of *MYCNUT* (G), *MYCNOS* (H), and *MYCN* in Day 8 EVT (*n* = 3 technical replicates). Data were represented as the mean ± SD. Data were analyzed by two-way ANOVA test (****P < 0.0001).

To further explore the potential regulatory relationships between lncRNAs and neighboring PCGs in the human placenta, we divided the genome into 5-kb windows and analyzed the coexpression patterns within these regions. Notably, the lncRNA–PCG pairs exhibited significant correlations in expression (Fig. S2A), suggesting potential *cis*-regulatory interactions. Moreover, we categorized lncRNA–PCG pairs in trophoblasts into six distinct locus configurations according to previous descriptions [9] (Fig. 2A). Among these, XH was the most frequent configuration (33.59%), whereas XO was the rarest (2.19%). The other configurations occurred at comparable frequencies, ranging from 12.08% to 19.98%. The occurrence of all six distributions in the extraembryonic lineage indicates a universal genomic organizational pattern. Notably, among these configurations, both antisense and sense lncRNAs were consistently positively correlated with the expression of their nearest PCGs (Fig. 2B); this correlation was further corroborated across three independent external datasets [33–35] (Fig. S2B–D, Table S1), revealing a pattern that differed from that observed in embryonic cells [14, 16].

Pseudotime reconstruction of the trophoblast lineage revealed three major trajectories during placental development: the maturation of CTB, differentiation from CTBs to EVTs, and differentiation from CTBs to STBs (Fig. S2E). Similarly, the expression dynamics of lncRNA–mRNA pairs with different genomic configurations tended to be positively correlated along these developmental trajectories (Fig. 2C). For example, the XH-type lncRNA *NR2F2*–*AS1*was highly expressed in CTBs with the self-renewal regulator *NR2F2* [36] (Fig. 2C), suggesting that *NR2F2*–*AS1* plays a role in regulating CTB stemness. In addition, during EVT differentiation, both XT-type *GATA2–AS1*–*GATA2* and SU-type *MYCNUT*–*MYCN* were upregulated, suggesting that lncRNAs and PCGs may synergistically contribute to trophoblast invasion [34, 37] (Fig. 2C). Moreover, the transmembrane protein *SEMA6A* and related XI-type *SEMA6A–AS1* (Fig. 2C), which were previously reported to exhibit an inverse expression relationship in hepatocellular carcinoma [38], were synchronously upregulated during STB differentiation, suggesting a specific interaction pattern during human trophoblast development.

We subsequently identified 21 high-confidence lncRNA–PCG regulatory pairs that were active during trophoblast developmental transitions (Fig. 2D), all of which were strongly positively correlated (Fig. S2F). To validate the potential *cis*-regulation acted by lncRNAs, we selected three lncRNAs that were active according to the three major trophoblast developmental trajectories discussed above (Fig. S2E) and explored the effects of lncRNA knockdown on the transcription of related PCGs using a well-established *in vitro* hTSC model (Fig. S3A). Consistent with their enrichment in CTB *in vivo* (Fig. 2D), the lncRNA *ECEL1P2* and its neighboring gene *ALPP* were actively co-expressed in *in vitro* hTSCs, distinguishing the trophoblast lineage from human embryonic stem cells (hESCs) (Fig. S3B). The knockdown of *ECEL1P2* led to decreased *ALPP* mRNA (Fig. 2E). In addition, the lncRNA *SEMA3B–AS1* and its neighboring gene *SEMA3B* were both upregulated during STB differentiation (Figs. 2D and S3C), and *SEMA3B–AS1* deficiency significantly downregulated the expression of *SEMA3B* (Fig. 2F). Interestingly, in addition to SU-type *MYCNUT*–*MYCN*, *MYCN* also showed a close relationship with another antisense lncRNA *MYCNOS*, and these genes were highly expressed during EVT differentiation (Figs. 2D and S3D). Consistently, both *MYCNUT* and *MYCNOS* deficiency impaired the expression of *MYCN* (Fig. 2G and 2H). Mechanistically, chromatin immuno­precipitation-qPCR (ChIP-qPCR) analysis revealed that knockdown of these lncRNAs significantly disrupted the local epigenetic landscape at their respective target loci (Fig. S4). The diverse and complex alterations observed among active (H3K9ac, H3K4me3) and repressive (H3K27me3) marks indicate that these lncRNAs are essential for organizing and maintaining locus-specific chromatin states, likely acting as critical structural or regulatory components within the local chromatin architecture. These findings provide epigenetic evidence for lncRNA-mediated *cis*-regulatory control through the remodeling of local histone signatures in the human placental trophoblast lineage.

### lncRNA deficiency impairs trophoblast self-renewal ability

Differential expression analysis revealed extensive lncRNA reprogramming during trophoblast development (Fig. S5A). Further functional enrichment analysis of the neighboring PCGs of these differential lncRNAs strongly linked them to placental developmental programs, including stemness maintenance regulation during CTB maturation, oxidative stress response during EVT differentiation, and metabolic regulation during STB differentiation (Fig. S5B). To evaluate their relevance to placental pathologies, we analyzed the expression of these lncRNA–PCG regulatory pairs in placental tissues from patients with pathological pregnancies, including those with PE and GDM [39, 40]. Compared with healthy controls, approximately one-quarter of these pairs exhibited significant dysregulation (Fig. S5C), highlighting the potential involvement of these lncRNA–PCG axes in placental development and pregnancy health.

**Figure 3.**
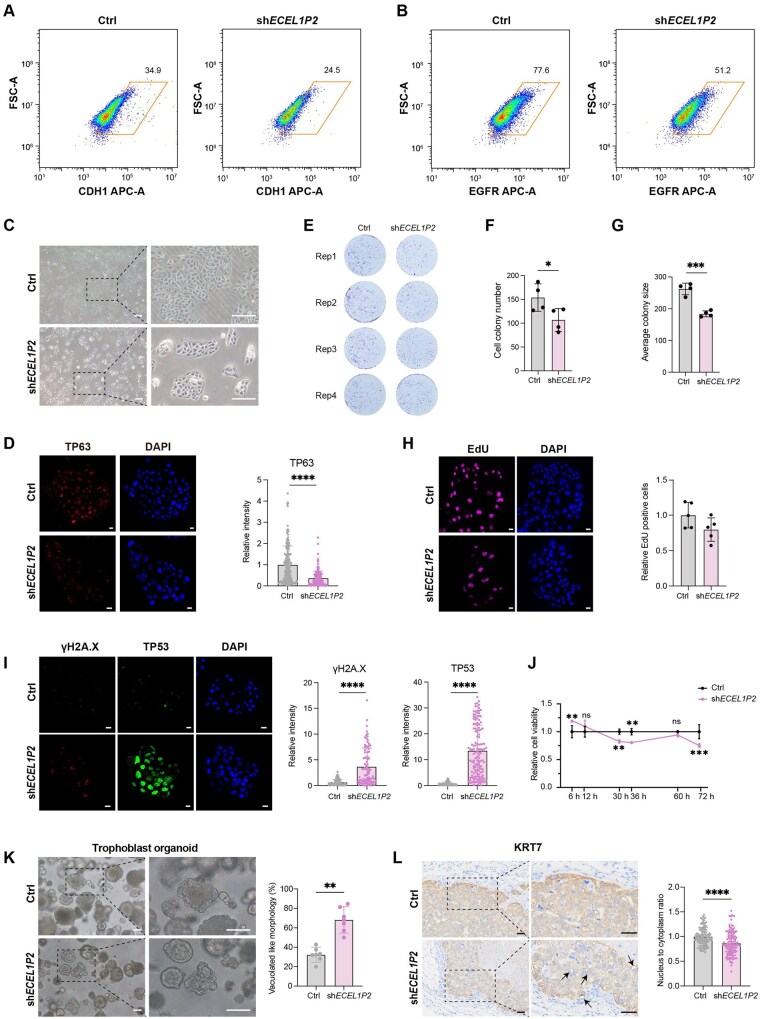
*ECEL1P2* depletion impairs the proliferative capacity of trophoblast stem cells. (A, B) Flow cytometric analysis of (A) CDH1 and (B) EGFR expression in hTSCs after *ECEL1P2* knockdown. (C) Bright-field images of hTSCs colonies following *ECEL1P2* knockdown. Scale bars = 250 μm. (D) Representative immunofluorescence images of TP63 (left) and quantitative analysis of its fluorescence intensity (right, *n* >140) in control and *ECEL1P2*-knockdown hTSCs. Data were represented as the mean ± SD. Data were analyzed by Student’s *t* test (****P < 0.0001). Scale bars = 20 μm. (E) Colony formation assay showing hTSCs proliferation rate after *ECEL1P2* knockdown. (F, G) Quantification of colony number (F) and colony size (G) formed in (E). Data were represented as the mean ± SD. Data were analyzed by two-way ANOVA test (***P < 0.001; *P < 0.05). (H) EdU incorporation assay. Left: Representative immunofluorescence images of EdU. Right: Quantification of the percentage of EdU-positive cells. Data were represented as the mean ± SD. Scale bars = 20 μm. (I) DNA damage and stress response analysis. Left: Representative immunofluorescence images of γH2A.X and TP53. Right: Quantitative analysis of the fluorescence intensity for γH2A.X (*n* > 70) and TP53 (*n* > 100). Data were represented as the mean ± SD. Data were analyzed by Student’s *t* test (****P < 0.0001). Scale bars = 20 μm. (J) Cell viability measured by CCK-8 assay at indicated time points (6–72 h) after *ECEL1P2* knockdown. Data were represented as the mean ± SD. Data were analyzed by two-way ANOVA test (***P < 0.001; **P < 0.01; ns: not significant). (K) Morphological analysis of trophoblast organoids. Left: Representative bright-field images of organoids derived from control and *ECEL1P2*-depleted hTSCs. Right: Quantitative analysis of the percentage of organoids exhibiting vacuolated-like morphology (*n* = 5 independent biological replicates). Data were represented as the mean ± SD. Data were analyzed by Student’s *t* test (**P < 0.01). Scale bars = 100 μm. (L) Immunohistochemical analysis of subcutaneous lesions. Left: Representative images of KRT7 staining in lesions formed by control and *ECEL1P2*-knockdown hTSCs in nude mice. Black arrows indicate vacuolated cells. Right: Quantitative analysis of the nucleus-to-cytoplasm ratio (*n* > 100). Data were represented as the mean ± SD. Data were analyzed by Student’s *t* test (****P < 0.0001). Scale bars = 50 μm.

To further determine and elucidate the regulatory roles of specific lncRNAs, we investigated the functional consequences of lncRNA deficiency using stem cell models (Fig. 2E–H). We first focused on *ECEL1P2*, an antisense lncRNA embedded within the *ALPP* locus and enriched in stem-like CTBs (Figs. 2D and S3B). While key transcriptional regulators such as *TFAP2C* and *ELF5* were not significantly affected (Fig. S6A), *ECEL1P2* knockdown led to the attenuation of epithelial traits, accompanied by loss of CDH1 and EGFR expression (Figs. 3A, 3B and S6B). In addition, *ECEL1P2* deficiency resulted in reduced hTSC proliferation (Fig. 3C), which coincided with the significant downregulation of *TP63* (Figs. 3D and S6B), a master regulator of CTB self-renewal [41]. Colony formation assay further confirmed a significant decline in both the number and size of colonies after *ECEL1P2* knockdown (Fig. 3E–G). This was further supported by decreased expression of the proliferation marker *MKI67* (Fig. S6C and S6D), indicating compromised self-renewal capacity.

To assess cell viability and elucidate mechanisms underlying growth defects, we performed further functional analyses of *ECEL1P2*-deficient hTSC. EdU incorporation assays validated the reduced proliferative activity (Figs. 3H and S6E), while cell cycle profiling revealed G0/G1 arrest and a decreased proportion of cells in the M phase (Fig. S6F). These changes were accompanied by upregulation of the cell cycle inhibitor *CDKN1C* and downregulation of CDK1, a key cell cycle promoter (Fig. S6G and S6H). These findings suggest a cell cycle blockade and aberrant cellular state. Further examination revealed elevated expression of TP53 and γH2A.X (Figs. 3I and S6I), marker of DNA damage and cellular stress. However, the stress response machinery appeared to be functionally impaired in *ECEL1P2*-deficient cells (Fig. S6J), suggesting a failure to adequately resolve stress and maintain homeostasis. This was supported by CCK-8 assays (Fig. 3J), which confirmed significantly reduced cell viability.

Notably, consistent with findings in 2D cultures, 3D TOs derived from *ECEL1P2*-deficient hTSCs exhibited severely impaired self-renewal, with fewer self-organized structures and the emergence of an abnormal vacuolated-like morphology (Figs. 3K and S6K). Moreover, the *in vivo* lesions from *ECEL1P2*-deficient hTSCs displayed prominent cytoplasmic vacuolization and nuclear pyknosis (Fig. 3L), suggesting increased cellular stress and apoptosis in a more physiological context. Together, these results suggest that *ECEL1P2* is a critical regulator of the self-renewal regulatory network in hTSC. Its deficiency disrupts cell cycle progression, compromises proliferative capacity, and triggers apoptotic responses, which ultimately impair trophoblast stem cell maintenance.

### Specific lncRNAs are essential for trophoblast differentiation

As *SEMA3B* and *MYCN* have been implicated in STB and EVT differentiation, respectively, we next investigated whether their associated regulatory lncRNAs influenced trophoblast lineage specification (Fig. 2D).

**Figure 4.**
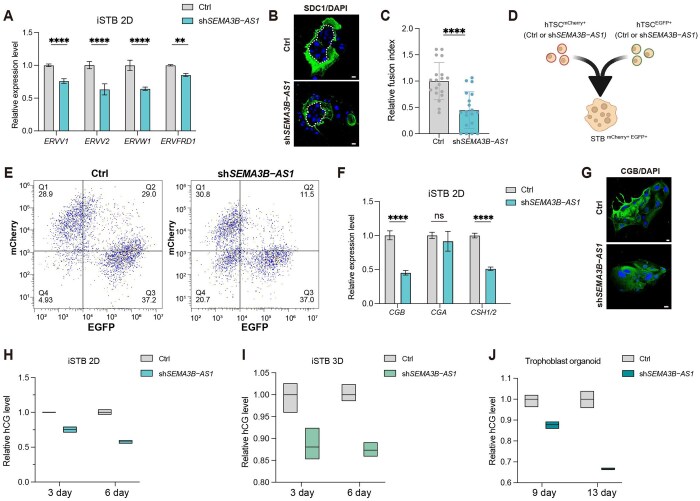
The lncRNA *SEMA3B*–*AS1* is required for syncytiotrophoblast differentiation and hormone production. (A) qRT-PCR analysis of syncytialization-related genes in 2D STBs after *SEMA3B–AS1* knockdown (*n* = 3 technical replicates). Data were represented as the mean ± SD. Data were analyzed by two-way ANOVA test (****P < 0.0001; **P < 0.01). (B) Immunofluorescence staining of SDC1 in 2D STBs following *SEMA3B–AS1* knockdown. Scale bars = 20 μm. (C) Quantification of the syncytialization index in 2D STBs after *SEMA3B–AS1* knockdown. Data were represented as the mean ± SD. Data were analyzed by Student’s *t* test (****P < 0.0001). (D) hTSC^mCherry+^ and hTSC^EGFP+^ transduced with Ctrl or sh*SEMA3B–AS1* were cocultured under STB differentiation conditions to generate fused STB^mCherry+/EGFP+^ cells. (E) Flow cytometric analysis of EGFP and mCherry fluorescence in 2D STBs after *SEMA3B–AS1* knockdown. (F) qRT-PCR analysis of pregnancy hormone genes in 2D STBs after *SEMA3B–AS1* knockdown (*n* = 3 technical replicates). Data were represented as the mean ± SD. Data were analyzed by two-way ANOVA test (****P < 0.0001; ns: not significant). (G) Immunofluorescence staining of CGB in 2D STBs following *SEMA3B–AS1* knockdown. Scale bars = 20 μm. (H, I) Secretion levels of hCG in 2D STBs (H) and 3D STBs (I) at Days 3 and 6 after *SEMA3B–AS1* knockdown (*n* = 3 technical replicates). (J) Secretion levels of hCG in trophoblast organoids at Days 9 and 13 after *SEMA3B–AS1* knockdown (*n* = 3 technical replicates).

During STB differentiation, knockdown of *SEMA3B–AS1* led to impaired syncytialization, characterized by the persistence of mononuclear cells with enlarged internuclear spaces resembling vacuoles (Fig. S7A). qRT-PCR analysis further supported this phenotype, showing significantly reduced expression of fusion-related genes (*ERVV1*, *ERVV2*, *ERVW1,* and *ERVFRD1*) (Fig. 4A), which are essential for the syncytialization process [37]. Consistently, immunofluorescence staining revealed a decrease in *SDC1* expression (Fig. 4B), and quantitative analysis confirmed a significant reduction in the formation of multinucleated syncytia (Fig. 4C). Using a dual-fluorescence reporter assay, we further validated the impaired syncytialization after *SEMA3B–AS1* knockdown (Figs. 4D, 4E and S7B). Moreover, knockdown of *SEMA3B–AS1* markedly compromised hormone-producing capacity (Fig. 4F and 4G). ELISA analysis further revealed an approximately 50% reduction in human chorionic gonadotropin (hCG) secretion (Fig. 4H). These findings were further corroborated by 3D STB differentiation models and TOs, both confirmed that loss of *SEMA3B–AS1* significantly impaired syncytialization and functional maturation of STBs (Figs. 4I, 4J and S7C–F). Together, these results highlight *SEMA3B–AS1* as a critical regulator of STB formation and endocrine function during placental development.

**Figure 5.**
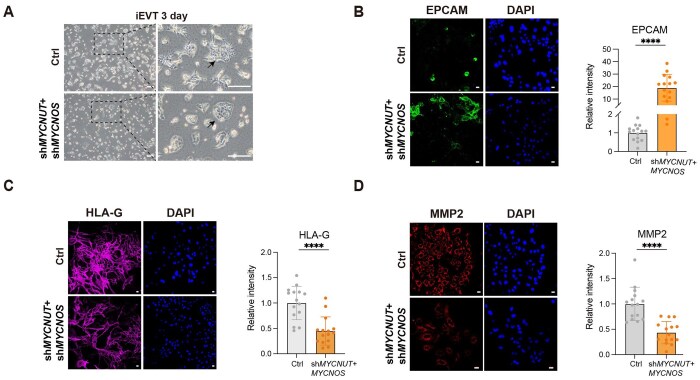
The lncRNA *MYCNUT* and *MYCNOS* regulate extravillous trophoblast differentiation. (A) Bright-field images of EVTs at Day 3 of differentiation. Scale bars = 250 μm. (B–D) Immunofluorescence staining and quantitative analysis of EPCAM (B), HLA-G (C), and MMP2 (D) in EVTs following *MYCNUT* and *MYCNOS* knockdown (*n* = 15). Data were represented as the mean ± SD. Data were analyzed by Student’s *t* test (****P < 0.0001). Scale bars = 20 μm.

During EVT differentiation, individual knockdown of *MYCNUT* or *MYCNOS* perturbed epithelial-to-mesenchymal transition (EMT) and downregulated the expression of canonical EVT markers (Fig. S8A and S8B). Interestingly, despite being transcribed in opposite orientations, knockdown of either lncRNA not only reduced the expression of the target gene *MYCN* but also altered the expression of the other lncRNA (Fig. S8C), suggesting they may exert similar functions in regulating EVT formation. To further investigate this potential synergy, we performed a double knockdown of both lncRNAs (Fig. S8D), which led to a consistent and significant delay in EMT during EVT differentiation (Fig. 5A). Notably, qRT-PCR and immunofluorescence staining revealed a failure to exit the epithelial state, as evidenced by sustained high levels of epithelial cell adhesion molecule (EPCAM) and cadherin-1 (E-cadherin) expression in the knockdown cells (Figs. 5B and S8E). Interestingly, although prolonged stimulation eventually induced an EVT-like morphology (Fig. S8F), functional maturation remained compromised. Specifically, expression of HLA-G, a key regulator of immune tolerance at the maternal–fetal interface, was markedly suppressed in the knockdown group (Fig. 5C). Moreover, MMP2, a crucial effector of cell invasion and migration, was greatly downregulated (Fig. 5D).

Collectively, these data demonstrate that the *SEMA3B–AS1*–*SEMA3B* and *MYCNUT*/*MYCNOS*–*MYCN* pairs play critical and distinct roles in regulating STB and EVT differentiation, respectively.

## Discussion

The placenta is essential for reproductive success, with the establishment of a robust trophoblast transcriptional program forming the foundation for its functional capacity. While previous studies have extensively characterized the roles of mRNAs and their encoded proteins in regulating trophoblast development, the contributions of noncoding RNAs, particularly lncRNAs, to human trophoblast fate and placental development remain elusive. Here, we integrated single-cell transcriptomic data to systematically characterized the dynamic expression landscape and functional regulatory networks of lncRNAs during human placental trophoblast development. Notably, given the predominant nuclear localization of lncRNAs, the use of snRNA-seq enabled more sensitive detection of these nuclear transcripts. Genomic colocalization analysis combined with functional validation revealed that lncRNAs frequently exert strong *cis*-activating effects on neighboring PCGs, and play critical roles in regulating human trophoblast self-renewal and lineage specification.

Emerging evidence suggests that lncRNAs often act as *cis*-elements that regulate adjacent PCGs through coexpression or mutual activation [9]. Consistently, we found that in human trophoblasts, lncRNAs are positively coexpressed with neighboring PCGs, regardless of their genomic configurations, trophoblast subtypes, and gestational stages. Moreover, both lncRNAs and mRNAs exhibited synchronous temporal expression pattern during trophoblast maturation and differentiation, suggesting coordinated regulation or functional coupling. These observations contrast with findings in preimplantation embryos, where lncRNAs are often activated prior to PCGs during zygotic genome activation (ZGA) [15]. The broader developmental span between early and late gestation in postimplantation trophoblasts may warrant finer temporal resolution in future studies.

Functionally, knockdown of specific lncRNAs in hTSCs resulted in reduced transcription of neighboring PCGs, implying that lncRNAs directly promote transcriptional activation rather than posttranscriptional regulation in this context. Prior studies have suggested that lncRNAs contribute to the organization of chromatin state and epigenomic remodeling [42, 43]. Consistent with this regulatory framework, we propose that the identified *cis*-activating lncRNAs such as *ECEL1P2* and *MYCNOS* may function as molecular scaffolds that recruit histone methyltransferases (e.g. MLL complex via WDR5 interaction) or acetyltransferases to deposit active marks (H3K4me3/H3K9ac) and facilitate enhancer–promoter communication [44–46]. Conversely, for the *SEMA3B–AS1* axis, the lncRNA likely operates via a “decoy” mechanism to antagonize the recruitment of Polycomb repressive complexes (PRC2), thereby preventing the deposition of silencing H3K27me3 marks [47, 48]. Our data suggest that these lncRNAs exert positive *cis*-regulatory effects, essential for maintaining chromatin state at target gene promoters. Notably, human trophoblasts are characterized by unique epigenetic features, including widespread partially methylated domains (PMDs) and a progressive global loss of histone modifications during gestation [49, 50]. In this context, it is plausible that these lineage-specific lncRNAs contribute to the local preservation of active chromatin states at essential developmental loci, thereby sustaining gene expression amidst the unique epigenetic landscape of the placenta.

Consistent with the notion that lncRNAs are highly cell-type-specific [37], our study revealed trophoblast subtype and gestational stage-dependent reprogramming of the lncRNA transcriptome. Functional studies involving lncRNA knockdown demonstrated that their biological significance in both trophoblast maturation and lineage differentiation. The strong positive *cis*-regulatory influence of lncRNAs suggested their involvement in human trophoblast lineage differentiation, usually mirroring the functions of their neighboring PCGs [8]. For example, *ALPP*, a placental alkaline phosphatase expressed on the fetal side of the placenta and essential for nutrient transfer [51], was downregulated following knockdown of its antisense lncRNA *ECEL1P2*, leading to impaired hTSC self-renewal. However, the precise contributions of PCG loss and the hierarchical regulatory relationships among lncRNAs, PCGs, and downstream effectors require further clarification. Moreover, recent studies have showed that lncRNAs with accessible chromatin states are enriched for binding sites of key trophoblast transcription factors, including GATA3, TEAD4, and TFAP2C [52], implicating a model in which these pioneer factors initiate chromatin remodeling, trigger lncRNA transcription, and modulate downstream gene networks. In line with this, a recent study proposed the existence of a *TP63*–*MYCNUT*–*MYCN* axis contributes to EVT differentiation [34], further emphasizing the intertwined roles of transcription factors and lncRNAs in coordinating the trophoblast developmental program.

**Figure 6.**
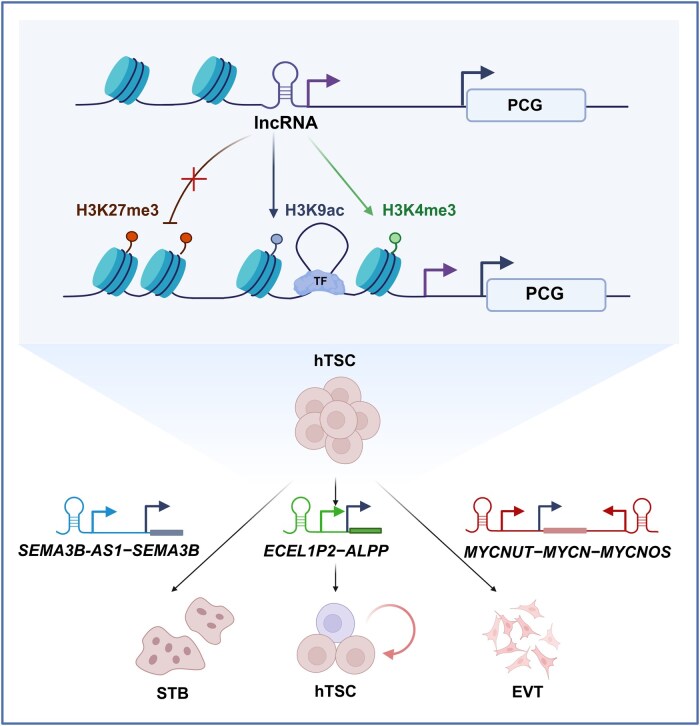
Schematic model of lncRNA-mediated *cis*-regulation driving trophoblast lineage specification. (Top) Lineage-specific lncRNAs act as structural scaffolds or decoys to maintain the epigenetic balance (H3K9ac/H3K4me3 vs. H3K27me3), thereby *cis-*regulating adjacent protein-coding genes (PCGs). (Bottom) Three distinct lncRNA–PCG axes regulate hTSC development: the *ECEL1P2*–*ALPP* axis maintains self-renewal, the *SEMA3B–AS1–SEMA3B* axis drives STB fusion, and the *MYCNUT/MYCNOS–MYCN* axis promotes EVT epithelial–mesenchymal transition.

In summary, our study characterizes the lineage-specific trajectories of lncRNA expression in human placental trophoblasts, and functionally demonstrates that lncRNAs serve as critical regulators of stemness maintenance and differentiation, largely by acting in *cis* to orchestrate promoter chromatin states of neighboring genes. The dysregulation of lncRNA–mRNA regulatory pairs in placental pathologies (PE and GDM) emphasizes the clinical relevance of these noncoding networks. Our findings offer new insight into the epigenetic and transcriptional control of human placental development, and suggest promising avenues for identifying novel biomarkers and therapeutic targets for pregnancy-associated disorders (Fig. 6).

### Research limitations

While this study provides a comprehensive analysis of the dynamic expression patterns and functional roles of lncRNAs in human placental trophoblast development, several limitations should be acknowledged. First, although our transcriptomic data are derived from *in vivo* placental tissues, the experimental validations were primarily performed using *in vitro* models, including hTSC and TOs. While these are well-established and physiologically relevant systems, they may not fully recapitulate the complexity of the *in vivo* microenvironment. Second, the lncRNA−PCG regulatory relationships identified here were largely inferred from coexpression and genomic proximity analyses. Third, our study primarily focused on *cis*-acting lncRNAs, leaving the potential roles of trans-acting lncRNAs or lncRNA–protein interactions largely unexplored. These aspects warrant further investigation to fully delineate the multilayered regulatory functions of lncRNAs in placental biology.

## Methods

### snRNA-seq processing and analysis

Raw snRNA-seq reads were processed with Cell Ranger (v8.0.1) using the GENCODE (v32) gene annotation. Downstream analyses were performed in R using Seurat (v5.1.0) Quality control followed Wang [27], retaining nuclei with ≥ 3,500 UMI counts and ≤ 50,000 UMIs, expressing 1,500–5,000 genes, and with mitochondrial gene content < 5%. Potential doublets were removed using Scrublet (v0.2.3) in Python with a threshold of 0.5.

Normalized expression matrices were used for feature selection and dimensionality reduction. The top 2,000 highly variable genes were selected for principal component analysis (PCA). Statistically significant principal components were determined via JackStraw analysis (500 replicates), and the top 30 PCs were used for subsequent analyses. Batch effects were corrected using Harmony (v1.2.3), followed by nonlinear dimensionality reduction with UMAP. Clustering resolution was optimized using clustree and set to 0.7. Cluster-specific marker genes were identified with Seurat’s FindAllMarkers function.

### Correlation analysis between lncRNAs and protein-coding genes

Correlation analysis between lncRNAs and PCGs in single-cell RNA-seq data was performed as follows. Single-cell expression matrices were first smoothed using the R package MAGIC (v3.0.0) to impute dropouts and reduce technical noise. Subsequently, Pearson correlation coefficients between each lncRNA and its respective PCGs were calculated using the R function cor.test ().

### Pseudotime trajectory analysis

Pseudotemporal ordering was performed using Monocle2 (v2.30.1). UMI count matrices of early trophoblast subtypes (early CTBs, STBs, EVTs, and late CTBs) extracted from Seurat objects were converted into Monocle2 CellDataSet objects. Genes expressed in fewer than 10 cells were excluded. Size factors and dispersion estimates were calculated using Monocle2 default settings. Ordering genes were selected based on Seurat differential expression results, including genes with log_2_ (fold change) ≥ 2, adjusted *P*-value < 0.05, and within the top 20% of expression. Both PCGs and lncRNAs were included. DDRTree (v0.1.5) was used for dimensionality reduction, and cells were ordered along developmental trajectories using the orderCells function.

### RNA velocity analysis

To estimate the transcriptional kinetics and directional flow of cell differentiation, RNA velocity analysis was performed using the velocyto and scVelo framework. First, spliced and unspliced expression matrices were generated from the Cell Ranger output BAM files using the velocyto command-line tool (v0.17.17) with the standard run10x logic. The resulting loom files were merged with the Seurat-processed metadata and converted into AnnData format for downstream analysis in Python using scVelo (v0.3.3). Genes were filtered based on minimum shared counts (min_shared_counts = 20) and top likelihood variability. The dynamical model was employed to solve the full transcriptional kinetics of splicing and degradation rates. Velocity vectors were projected onto the pre-computed UMAP embedding. Velocity length—quantified as the length of the velocity vector in the high-dimensional gene expression space—was calculated to reflect the rate of differentiation and transcriptional activity of individual cells.

### GO enrichment analysis

Functional enrichment analysis was performed with ClusterProfiler (v4.10.1).

### Bulk RNA-seq processing and differential expression analysis

The transcriptional expression matrix was obtained from Okae [18]. Normalization and differential expression analyses were performed using DESeq2 (v1.42.1).

### Experimental model and study participant details

Specific-pathogen-free (SPF) BALB/c nude mice were maintained within the animal facility at Tongji University (Shanghai, China). The animals were housed under standard environmental conditions, which included a 12 h light/12 h dark cycle, and were provided *ad libitum* access to standard chow and water.

### Culture of hTSC

hTSC were generated following the published protocol [18]. Briefly, a 35 mm dish was coated with 5 μg/mL collagen IV (Sigma-Aldrich, C7521) at 37 °C for at least one hour and a density of 8 × 10^4^–10 × 10^4^ cells were seeded onto the precoated dish. Cells were then cultured in 2 mL of standard TS medium: DMEM/F12 (Thermo Fisher Scientific, 11320033) supplemented with 0.1 mM 2-mercaptoethanol (Thermo Fisher Scientific, 21-985-023), 0.2% Fetal Bovine Serum (FBS) (Gibco, 16000-044), 0.5% Penicillin-Streptomycin (Thermo Fisher Scientific, 15140163), 0.3% bovine serum albumin (BSA) (Bovine Albumin, 199896), 1% ITS-X supplement (BasalMedia, S452J7), 1.5 μg/mL L-ascorbic acid (Sigma-Aldrich, A4544), 50 ng/mL EGF (PeproTech, AF-100-15), 2 μM CHIR99021 (Selleck Chemicals, S2924), 0.5 μM A83-01 (Selleck Chemicals, S7692), 1 μM SB431542 (Selleck Chemicals, S1067), 0.8 mM valproic acid (VPA) (Sigma-Aldrich, PHR1061-1G), and 5 μM Y27632 (Selleck Chemicals, S1049) at 37 °C in 5% O_2_. When cells reached about 80% confluence, they were dissociated with TrypLE (Thermo Fisher Scientific, 12604021) for 10–15 min at 37 °C and passaged at 1:3–1:4 split ratio. The TSC medium was replaced every day.

### Plasmid and cell line construction

To construct hTSC-membrane-EGFP/mCherry (hTSC^EGFP+^/hTSC^mCherry+^) cell lines, the plasmids FUGW-membrane-EGFP or FUGW-membrane-mCherry were introduced by lentivirus infection. After 48 h of infection, the EGFP- or mCherry-positive cells were sorted and collected for further culture.

To establish stable lncRNA-knockdown hTSC lines, we utilized the pLKO.1-TRC lentiviral vector system. The plasmid was linearized by double digestion with AgeI (New England Biolabs, R3552L)/EcoRI (New England Biolabs, R3101S) restriction endonuclease and purified for subsequent cloning. Using an online website DSIR (biodev.extra.cea.fr/DSIR/DSIR.html) to design multiple shRNA sequence targets, annealed to synthesize oligonucleotides, and connected to the pLKO.1-TRC vector. For lentiviral production, the constructed shRNA plasmids were co-transfected with psPAX2 and pMD2.G into HEK293T cells using Vigofect (Vigorous, T001) transfection reagent, following the manufacturer’s protocol. Viral supernatants were collected at 48–72 h posttransfection, filtered (0.45 μM), and concentrated by centrifugation (4800 rpm, 30 min, 4 °C). For hTSC infection, cells were seeded at about 50% confluency and transduced with shRNA lentivirus for 6–8 h. Then the medium was replaced, and 1 μg/mL puromycin was added for stable selection. An empty pLKO.1-TRC vector was used as a negative control to account for off-target effects. The sequences of the oligonucleotides are provided in Table S2.

### Differentiation of hTSC

Differentiation of hTSC toward EVT and STB was performed as previously described [18]. Briefly, for induction of EVT (iEVT), a density of 7.5 × 10^4^ hTSC was seeded in a 35 mm dish precoated with 1 μg/mL collagen IV. Cells were cultured in 2 mL of EVT medium [DMEM/F12 supplemented with 0.1 mM 2-mercaptoethanol, 0.5% Penicillin–Streptomycin, 0.3% BSA, 1% ITS-X supplement, 100 ng/mL NRG1 (Sino Biological, 13499-H08H-100), 7.5 mM A83-01, 2.5 mM Y27632, and 4% KnockOut Serum Replacement (KSR) (Thermo Fisher Scientific, 10828028)], and Matrigel (Corning, 356231) was added to the medium containing the cells at a final concentration of 2%. On the third day, replace the fresh EVT medium without NRG1 and add Matrigel to a final concentration of 0.5%. On the sixth day, the cells were reached about 80% confluence, they were dissociated with TrypLE for 15–20 min at 37 °C and passaged at 1:2 split ratio. Suspended the cells with EVT medium lacking NRG1 and KSR, add Matrigel to a final concentration of 0.5%, and cultured for another two days.

For induction of 2D-STB (iSTB-2D), a density of 1 × 10^5^ hTSC was seeded in a 35 mm dish precoated with 2.5 μg/mL collagen IV. Cells were cultured in 2 mL of STB-2D medium [DMEM/F12 supplemented with 0.1 mM 2-mercaptoethanol, 0.5% Penicillin–Streptomycin, 0.3% BSA, 1% ITS-X supplement, 2.5 μM Y27632, 2 μM forskolin (Selleck Chemicals, S2449), and 4% KSR]. The STB-2D medium was replaced at Day 3, and the cells were collected at Day 6 for analysis.

For induction of 3D-STB (iSTB-3D), a density of 2.5 × 10^5^ hTSC was seeded in a 6 cm Petri dish. Cells were cultured in 3 mL of STB-3D medium [DMEM/F12 supplemented with 0.1 mM 2-mercaptoethanol, 0.5% Penicillin–Streptomycin, 0.3% BSA, 1% ITS-X supplement, 2.5 μM Y27632, 50 ng/mL EGF, 2 μM forskolin, and 4% KSR]. After 3 days of culture, add an equal amount of fresh STB-3D medium into the dish. The cells were collected by using 40 μM filter to remove dead cells at Day 6. Both iEVT and iSTB were cultured under the condition of 37 °C and 5% O_2_.

To quantitatively assess cell fusion and STB formation, 1 × 10^5^ hTSC^EGFP+^ and 1 × 10^5^ hTSC^mCherry+^ cells were thoroughly mixed and co-seeded into a 35 mm dish containing STB-2D differentiation medium. The medium was replaced with fresh media after 24 h. After further culture 48 h, cells were dissociated with TrypLE for 15–25 min and analyzed by flow cytometry. The double-positive cells were defined as fused STBs.

### TOs formation and cultivation

For the generation of TOs, cells were dissociated with TrypLE for 10–15 min and collected by centrifugation at 1100 rpm for 4 min. Subsequently, 3 × 10³ cells were resuspended in 25 μL of ice-cold Matrigel and mixed gently. Pipette the drop into the center of each well of a 48-well tissue culture plate and place in the incubator to set for at least 15 min. TOs were cultured in 250 μL of TO medium (TOM): Advanced DMEM/F12 (Thermo Fisher Scientific, 12634010), 1×N2 (Invitrogen, 17502048), 1×B27-VA (Thermo Scientific, 12587010), 1.25 mM N-acetyl-L-cysteine (Sigma-Aldrich, A9165), 2 mM L-glutamine (Millipore Sigma, TMS-002-C), 50 ng/mL EGF, 1.5 μM CHIR99021, 80 ng/mL Recombinant human R-spondin-1 (PeproTech, 120-38), 100 ng/mL Recombinant human FGF-2 (PeproTech, 100-18B-50), 50 ng/mL HGF (Sino Biological, 10463-HNAS-100), 500 nM A83-01, 2.5 μM prostaglandin E2 (Selleck, S3003), 2 μM Y27632, 100 μg/mL primocin (Invivogen, ANT-PM-1). The TOM was replaced every 3 days.

### Measurement of hCG

To measure hCG concentration in the differentiation of iSTB-2D and iSTB-3D, the medium was collected at Days 3 and 6. To measure hCG concentration in the TO formation, the medium was collected at Days 9 and 13. Then the medium was centrifuged at 1100 rpm for 5 min to remove cell debris. The supernatant was harvested and then they were processed for hCG level by Shanghai Enzyme-Linked Biotechnology Co., Ltd.

### Cell proliferation assay

Cell growth and viability were evaluated utilizing the Cell Counting Kit-8 (CCK-8). Cells were plated into 96-well microtiter plates at a density of 2 × 10^3^ cells per well and cultured over consecutive days. At a consistent time point each day, 10 μL of the CCK-8 reagent (MCE, HY-K0301) was introduced into each well, followed by a 2 h incubation period. Absorbance was subsequently measured at 450 nm using a Spark^®^ microplate reader (Tecan Trading AG, Switzerland) to quantify cell viability.

### Flow cytometry

For epithelial characteristics markers analysis, cells were dissociated by TrypLE and treated with APC-conjugated anti-CDH1 antibody (BioLegend, 147311) or APC-conjugated anti-EGFR antibody (BioLegend, 352906) for 30 min on ice. Then washed with Dulbecco’s phosphate-buffered saline (DPBS) (Servicebio, G4200) containing 1% BSA for three times and determined by CytoFLEX S (Beckman Coulter, USA).

### Cell cycle assay

Cell cycle assay was performed using propidium iodide (PI) (Beyotime, ST511) in accordance with the manufacturer’s instruction. Briefly, harvested cells were fixed overnight in 70% ethanol at 4 °C. After fixation, the cells were washed three times with DPBS and subsequently stained in the dark with a solution containing RNase A (500 μg/mL) (TIANGEN, RT405) and PI (50 μg/mL) for 30 min at 37 °C. Following staining, the cells were centrifuged at 1100 rpm for 5 min, resuspended in DPBS, and subjected to flow cytometry analysis.

### Colony formation assay

hTSC were seeded in 12-well plates precoated with 7.5 μg/mL collagen IV (5 × 10^3^ cells per well) and cultured for 96 h. After the supernatant was removed and gently washed three times with DPBS, the cells were fixed with formaldehyde for 10 min. Subsequently, the cells were stained with crystal violet (Beyotime, C0121) for 10 min and washed with DPBS. After natural air-drying, the culture plate was scanned, the colony number and colony size were calculated using ImageJ.

### EdU assay

EdU (10 μM) was added to the medium during the period of vigorous cell division and treated for 2 h. Then cells were harvested for fixation, permeabilization, and the Click reaction using Azide 647 (Beyotime, C0081S). 4′,6-diamidino-2-phenylindole (DAPI) (Beyotime, C1002) was used for DNA count according to the manufacturer’s recommendations. EdU-positive cells were quantified by confocal microscopy (ZEISS LSM880) or flow cytometry.

### Quantitative RT-PCR

Total RNA was isolated using RNAiso plus (Takara, 9109) and reverse-transcribed using 5× All-In-One RT Master Mix (ABM, G490) according to the manufacturer’s recommendations. Quantitative RT-PCR was performed using SYBR Premix Ex Taq II (Takara, RR820B) and signals were detected with QuantStudio3 (Thermo Fisher Scientific, A28136). Data were normalized to *GAPDH* expression with 2^−ΔΔCT^ method (*n *= 3). Details of the primers are provided in Table S2.

### Immunofluorescence

Cells were seeded on Matrigel-coated glass slides and fixed with 4% paraformaldehyde (PFA) (Servicebio, G1101) for 10 min at room temperature. After three times wash with TBST (Epizyme, PS103), cells were permeabilized with 0.5% Triton X-100 (Sigma-Aldrich, T8532) for 15 min. The samples were blocked with 2.5% BSA at 25 °C for 1 h and then incubated overnight at 4 °C with the following primary antibodies: TP53 (Santa Cruz, sc126), γH2A.X (Abcam, ab81299), Ki67 (Cell Signaling Technology, 9129 T), TP63 (Abcam, ab124762), SDC1 (Proteintech, 67155-1-Ig), CGB (Abcam, ab9582), E-Cadherin (Cell Signaling Technology, 31955), HLA-G (Abcam, ab52454), MMP2 (Cell Signaling Technology, 40994), or EPCAM (Invitrogen, MA512436). Cells were then washed three times with TBST and incubated with secondary antibodies for 1 h at room temperature. The nuclei were stained with DAPI. Confocal imaging was performed with Zeiss LSM 880 confocal microscopes and analyzed with Zeiss Zen Blue Edition.

### Western blot

Cells were lysed in RIPA lysis buffer (Beyotime, P0013B) with protease inhibitor cocktail (Roche, 04693132001), then denatured in loading buffer by heating at 95 °C for 10 min. Denature protein were separated by 10% SDS–PAGE gels (Epizyme, PG112) in Tris-glycine running buffer (Epizyme, PS105) and transferred onto PVDF membranes (Millipore Sigma, IPVH00010). The membrane was blocked with 5% BSA in TBST for 1 h at room temperature and then incubated overnight at 4 °C with the following primary antibodies: CDK1 (Santa Cruz, sc-54), GAPDH (Proteintech, 60004). Following extensive washes with TBST, the membrane was incubated with an HRP-conjugated goat anti-mouse IgG secondary antibody (Epizyme, LF101) for 1 h at room temperature. Signal detection was performed using a SuperSignal West Pico PLUS (Thermo Scientific, 34580) and visualized with a ChemiDoc MP Imaging System. The intensity of the bands was quantified using ImageJ software.

### Engraftment of hTSC into BALB/C nude mice

5 × 10^6^ hTSC were dissociated with TrypLE for 10–15 min and resuspended in 100 μL of a mixture (Matrigel and DMEM/F12 containing 0.3% BSA and 1% ITS-X supplement at 1:2 split ratio). The suspensions were subcutaneously injected into 6- to 8-week-old male BALB/C Nude mice. Lesions were collected a week after injection and fixed with 4% PFA overnight at 4 °C. The lesions were sent to Wuhan Servicebio Technology Co., Ltd for anti-KRT7 (Abcam, ab68459) immunohistochemistry and scanned with Olympus VS200.

### Chromatin immunoprecipitation-qPCR

Following a previously established protocol [53]. ChIP-qPCR assays were conducted utilizing 1 × 10^4^ cells per reaction. Briefly, harvested cells were resuspended in a nuclear extraction buffer, and the chromatin was sheared by treatment with MNase for 10 min at 25 °C. The sheared chromatin samples were then subjected to overnight immunoprecipitation at 4 °C with 1 μg of specific antibodies conjugated to 10 μL of Protein A Dynabeads (Life Technologies). Both the immunoprecipitated (IP) DNA and input DNA were isolated via phenol–chloroform extraction and subsequent ethanol precipitation. Prior to quantitative PCR analysis, the purified DNA was diluted 3-fold. The following primary antibodies were utilized: anti-IgG (Vazyme, Ab207-01), anti-H3K4me3 (Cell Signaling Technology, 9751S), anti-H3K27me3 (Diagenode, C15410195), and anti-H3K9ac (Abcam, ab4441). All primer sequences employed for this assay are provided in Table S2.

### Research ethics

All *in vivo* animal procedures and husbandry practices adhered strictly to the Health Guide for the Care and Use of Laboratory Animals. The animal experimental protocols were formally reviewed and approved by the Biological Research Ethics Committee of Tongji University.

The collection of human placental tissue for hTSC derivation was approved by the Ethics Committee of Shanghai First Maternity and Infant Hospital (Approval No. KS23268). Written informed consent was obtained from all enrolled subjects prior to their participation, and the study was conducted in accordance with the principles of the Declaration of Helsinki.

### Statistical analysis

Statistical analyses were performed using GraphPad Prism (version 10.3.0) or R (version 4.3.1). Data are presented as mean ± SD. Group comparisons were conducted using Student’s *t*-test or two-way ANOVA, while Wilcoxon rank-sum tests were applied for snRNA-seq data. Sample sizes are detailed in the figure legends. *P *< 0.05 was considered statistically significant (*P < 0.05, **P < 0.01, ***P < 0.001, ****P < 0.0001; ns, not significant).

## Supplementary Material

lnag010_Supplementary_Data

## Data Availability

The single-nucleus RNA-seq datasets analyzed in this study were obtained from the Gene Expression Omnibus (GEO) under accession number GSE247038. For cross-platform validation: Three independent external datasets were utilized, including two single-cell RNA-seq datasets (accession numbers: HRA003309, GSE214607) and one single-nucleus RNA-seq dataset (singlecell.broadinstitute.org/single_cell/study/SCP2601). The transcriptomic data of early trophoblast stem cells were retrieved from the supplementary materials of Okae et al. (Cell Stem Cell, 2018) [19]. Placental datasets from pathological pregnancies, including preeclampsia and gestational diabetes mellitus, were obtained from GSE173193. All other data supporting the findings of this study are available from the corresponding author upon reasonable request.
