# Supplementary material for: Single-cell analysis reveals essential lncRNAs regulating human trophoblast lineage differentiation
Source: Life Med. 2026 Apr 17;5(2):lnag010. doi: 10.1093/lifemedi/lnag010 (PMC13117612; doi:10.1093/lifemedi/lnag010)
Supplement: lnag010_Supplementary_Data [file lnag010_supplementary_data.zip › 08-Apr-2026_095208_Supplementary_Material.zip!/Supplementary_Material/SI_UPLOAD.docx]

**Single-cell Analysis Reveals Essential lncRNAs Regulating Human Trophoblast Lineage Differentiation**

Weiyi Zhang^1,#^, Chunyan Le^1,#^, Shanru Yi^1,#^, Wenqiang Liu^1^, Xiaoyu Liu^1^, Zhen Yin ^2,3^, Hong Wang^1^, Rui Gao^1,*^, Shaorong Gao^1,*^, Jiayu Chen^1,*^

^1^Clinical and Translation Research Center of Shanghai First Maternity & Infant Hospital, Shanghai Key Laboratory of Signaling and Disease Research, Frontier Science Center for Stem Cell Research, School of Life Sciences and Technology, Tongji University, Shanghai 200092, China

^2^Department of Control Science and Engineering, State Key Laboratory of Autonomous Intelligent Unmanned Systems, Tongji University, Shanghai 201210, China

^3^Shanghai Innovation Institute, Shanghai 200030, China

^#^These authors contributed equally to this work.

^*^Correspondence: gaorui@tongji.edu.cn (R.G.), [gaoshaorong@tongji.edu.cn](mailto:gaoshaorong@tongji.edu.cn) (S.G.) [chenjiayu@tongji.edu.cn](mailto:chenjiayu@tongji.edu.cn) (J.C.)

**Extended Data Figure legends**

**Extended Data Figure S1. Quality control and overview of lncRNA expression in trophoblast snRNA-seq.**

(A) Feature counts versus mitochondrial transcript percentage across nuclei. (B) UMAP visualization colored by cell-cycle phase based on cell-cycle gene signatures. (C) UMAP feature plots showing the expression distribution of representative marker genes for each cell type (Color gradient represents the average expression level per cell). (D) Bar plot of cell number per cluster. (E) Pie plots of the fraction of lncRNAs (light) and mRNAs (dark) across trophoblast subtypes. The value “n” indicates the average number of detected transcripts per cell for both lncRNAs and mRNAs, calculated separately for each individual cell type. (F) Assessment of transcriptional kinetics via RNA velocity. Quantitative comparison of velocity length in the corresponding cell types (CTB, EVT, STB) between early and late gestation. Statistical significance was determined by two-sided Wilcoxon rank-sum tests (****P* < 0.001, *****P* < 0.0001). Late EVTs were excluded from the velocity analysis as their insufficient cell numbers precluded robust vector modeling. (G) Line plots of cell type-specific lncRNAs showing relative expression levels across trophoblast lineages (Green shows the expression level of individual lncRNAs; purple denotes the median expression of all lncRNAs). (H) Heatmap of representative lncRNAs and their neighboring PCGs across trophoblast subtypes.

**Extended Data Figure S2. Additional analyses of lncRNA–PCG regulation.**

(A) Genomic distribution of lncRNAs relative to neighboring protein-coding genes within ± 5 kb and pairwise expression correlations. (B–D) Violin plots illustrating the expression correlation between candidate lncRNAs and their adjacent mRNAs across trophoblast subtypes (CTB, EVT, STB) in three independent cohorts. (B) Data derived from early- and second-trimester samples ^33^. (C) Data sourced from a first-trimester spatial multi-omics atlas ^34^. (D) Data extracted from healthy controls (early gestation) in a recurrent miscarriage study ^35^. (E) Pseudotime trajectories of early CTBs, EVTs, STBs, and late CTBs. (F) Correlation strength between lncRNAs and PCGs across configurations.

**Extended Data Figure S3. Experimental design and expression validation of candidate lncRNA–PCG pairs during hTSC differentiation.**
(A) Experimental design of hTSC differentiation into STBs and EVTs. This figure was created using BioRender. (B-D) Left: Genomic localization and design of specific shRNA target sites for the selected lncRNA–PCG pairs. Right: qRT-PCR analysis of the indicated lncRNA–PCG pairs across trophoblast differentiation processes. Expression of each lncRNA and its neighboring protein-coding gene was quantified in hESCs and hTSCs (B), hTSCs and iSTBs (C), and hTSCs and iEVTs at different differentiation stages (day 3, day 6, and day 8) (D), respectively (*n* = 3 technical replicates). Data were represented as the mean ± SD. Data were analyzed by two-way ANOVA test (*****P* < 0.0001; ***P* < 0.01; ns: not significant).

**Extended Data Figure S4. Epigenetic regulation of target gene promoters by lineage-specific lncRNAs during trophoblast development.**

(A, B) ChIP-qPCR showing the enrichment of H3K9ac, H3K27me3, and H3K4me3 at two distinct regions (region 1 and region 2) of the *ALPP* promoter following *ECEL1P2* knockdown. Data were analyzed by Student’s *t* test (*****P* < 0.0001, **P* < 0.05). (C, D) ChIP-qPCR showing the enrichment of H3K9ac, H3K27me3, and H3K4me3 at region 1 and region 2 of the *SEMA3B* promoter after *SEMA3B-AS1* knockdown. Data were analyzed by Student’s *t* test (***P* < 0.01, **P* < 0.05). (E, F) ChIP-qPCR showing the enrichment of H3K9ac, H3K27me3, and H3K4me3 at region 1 and region 2 of the *MYCN* promoter upon *MYCNUT* knockdown. Data were analyzed by Student’s *t* test (***P* < 0.01, **P* < 0.05). (G, H) ChIP-qPCR showing the enrichment of H3K9ac, H3K27me3, and H3K4me3 at region 2 (shared with *MYCNUT*) and region 3 of the *MYCN* promoter following *MYCNOS* knockdown. Data were analyzed by Student’s *t* test (***P* < 0.01, **P* < 0.05), and the specific genomic coordinates for each gene promoter region are listed in the Table S2.

**Extended Data Figure S5. Bioinformatic analysis and clinical relevance of lncRNAs in trophoblast differentiation.**

(A) Differential expression profiles of lncRNAs across trophoblast differentiation stages: early CTBs, late CTBs, early EVTs, and early STBs. (B) Pathway enrichment analysis of biological processes associated with the distinct trophoblast differentiation stages. (C) Expression levels of lncRNA–mRNA pairs in placental tissues from patients with preeclampsia (PE) and gestational diabetes mellitus (GDM) compared to healthy controls.

**Extended Data Figure S6. Molecular consequences of ECEL1P2 knockdown in hTSCs.**

(A) qRT-PCR analysis of key trophoblast transcription factors after *ECEL1P2* knockdown. Data were represented as the mean ± SD. Data were analyzed by two-way ANOVA test (*****P* < 0.0001; ns: not significant). (B) qRT-PCR analysis of stemness maintenance and proliferation markers upon *ECEL1P2* knockdown (*n* = 3 technical replicates). Data were represented as the mean ± SD. Data were analyzed by two-way ANOVA test (*****P* < 0.0001; ****P* < 0.001).

(C) qRT-PCR analysis of the proliferation marker *MKI67* after *ECEL1P2* knockdown (*n* = 3 technical replicates). Data were represented as the mean ± SD. Data were analyzed by two-way ANOVA test (**P* < 0.05). (D) MKI67 immunofluorescence staining after *ECEL1P2* knockdown. Scale bars = 20 μm. (E) Flow cytometry histograms of EdU incorporation showing DNA synthesis after *ECEL1P2* knockdown. (F) Cell cycle distribution analyzed by flow cytometry in hTSCs after *ECEL1P2* knockdown (*n* = 3 technical replicates). Data were represented as the mean ± SD. Data were analyzed by two-way ANOVA test (*****P* < 0.0001; ns: not significant). (G) qRT-PCR analysis of *CDKN1C* following *ECEL1P2* knockdown (*n* = 3 technical replicates). Data were represented as the mean ± SD. Data were analyzed by two-way ANOVA test (*****P* < 0.0001). (H) Western blot analysis of protein expression levels for CDK1 in control and *ECEL1P2*-knockdown hTSCs. (I) qRT-PCR analysis of the *TP53* after *ECEL1P2* knockdown (*n* = 3 technical replicates). Data were represented as the mean ± SD. Data were analyzed by two-way ANOVA test (*****P* < 0.0001). (J) qRT-PCR analysis of the *PLAC8*, *FOS* and *OPTN* upon *ECEL1P2* knockdown (*n* = 3 technical replicates). Data were represented as the mean ± SD. Data were analyzed by two-way ANOVA test (*****P* < 0.0001; **P* < 0.05). (K) Representative morphology of *ECEL1P2*-knockdown trophoblast organoids. Bright-field images of organoids derived from control and *ECEL1P2*-depleted hTSCs. Scale bars = 100 μm.

**Extended Data Figure S7. SEMA3B-AS1 is critical for syncytiotrophoblast formation in 3D culture.**

(A) Bright-field images of 2D STBs at day 6 of differentiation following *SEMA3B-AS1* knockdown. Scale bars = 250 μm. (B) Representative fluorescence images from a dual-reporter (EGFP/mCherry) assay monitoring cell fusion in 2D STBs with *SEMA3B-AS1* knockdown. Scale bars = 20 μm. (C) Knockdown efficiency of *SEMA3B-AS1* in 3D STBs (*n* = 3 technical replicates). Data were represented as the mean ± SD. Data were analyzed by two-way ANOVA test (*****P* < 0.0001; ****P* < 0.001). (D) Left: Bright-field images of 3D STBs after *SEMA3B-AS*1 knockdown. Right: Quantification of syncytium area. Data were represented as the mean ± SD. Scale bars = 200 μm. (E) qRT-PCR analysis of fusion-related genes after *SEMA3B-AS1* knockdown (*n* = 3 technical replicates). Data were represented as the mean ± SD. Data were analyzed by two-way ANOVA test (*****P* < 0.0001; ****P* < 0.001; ***P* < 0.01). (F) qRT-PCR analysis of pregnancy hormone genes in 3D STBs after *SEMA3B-AS1* knockdown (*n* = 3 technical replicates). Data were represented as the mean ± SD. Data were analyzed by two-way ANOVA test (*****P* < 0.0001).

**Extended Data Figure S8. Functional interplay between MYCNUT and MYCNOS during EVT differentiation.**

(A) Bright-field images of EVTs at day 3 of differentiation after knockdown of *MYCNUT* or *MYCNOS*. Scale bars = 250 μm. (B) qRT-PCR analysis of EVT markers after individual knockdown of *MYCNUT* or *MYCNOS* at day 8 of EVT differentiation (*n* = 3 technical replicates). Data were represented as the mean ± SD. Data were analyzed by two-way ANOVA test (*****P* < 0.0001; ns: not significant). (C) qRT-PCR analysis of *MYCNUT*, *MYCNOS*, and *MYCN* after *MYCNUT* knockdown (Left) or *MYCNOS* (Right) knockdown at day 8 of EVT differentiation (*n* = 3 technical replicates). Data were represented as the mean ± SD. Data were analyzed by two-way ANOVA test (*****P* < 0.0001). (D) Expression dynamics of *MYCNUT*, *MYCNOS*, and *MYCN* in hTSC, iEVT-day 3, and iEVT-day 6 after combined knockdown of *MYCNUT* and *MYCNOS* (*n* = 3 technical replicates). Data were represented as the mean ± SD. Data were analyzed by two-way ANOVA test (*****P* < 0.0001). (E) Immunofluorescence staining of E-cadherin at day 6 of EVT differentiation after *MYCNUT* and *MYCNOS* knockdown. Scale bars = 20 μm. (F) Bright-field images of EVTs at day 3 of differentiation after combined knockdown of *MYCNUT* and *MYCNOS*. Scale bars = 500 μm.
