# Supplementary material for: Single-cell analysis reveals essential lncRNAs regulating human trophoblast lineage differentiation
Source: Life Med. 2026 Apr 17;5(2):lnag010. doi: 10.1093/lifemedi/lnag010 (PMC13117612; doi:10.1093/lifemedi/lnag010)

Extended Data Fig. S3

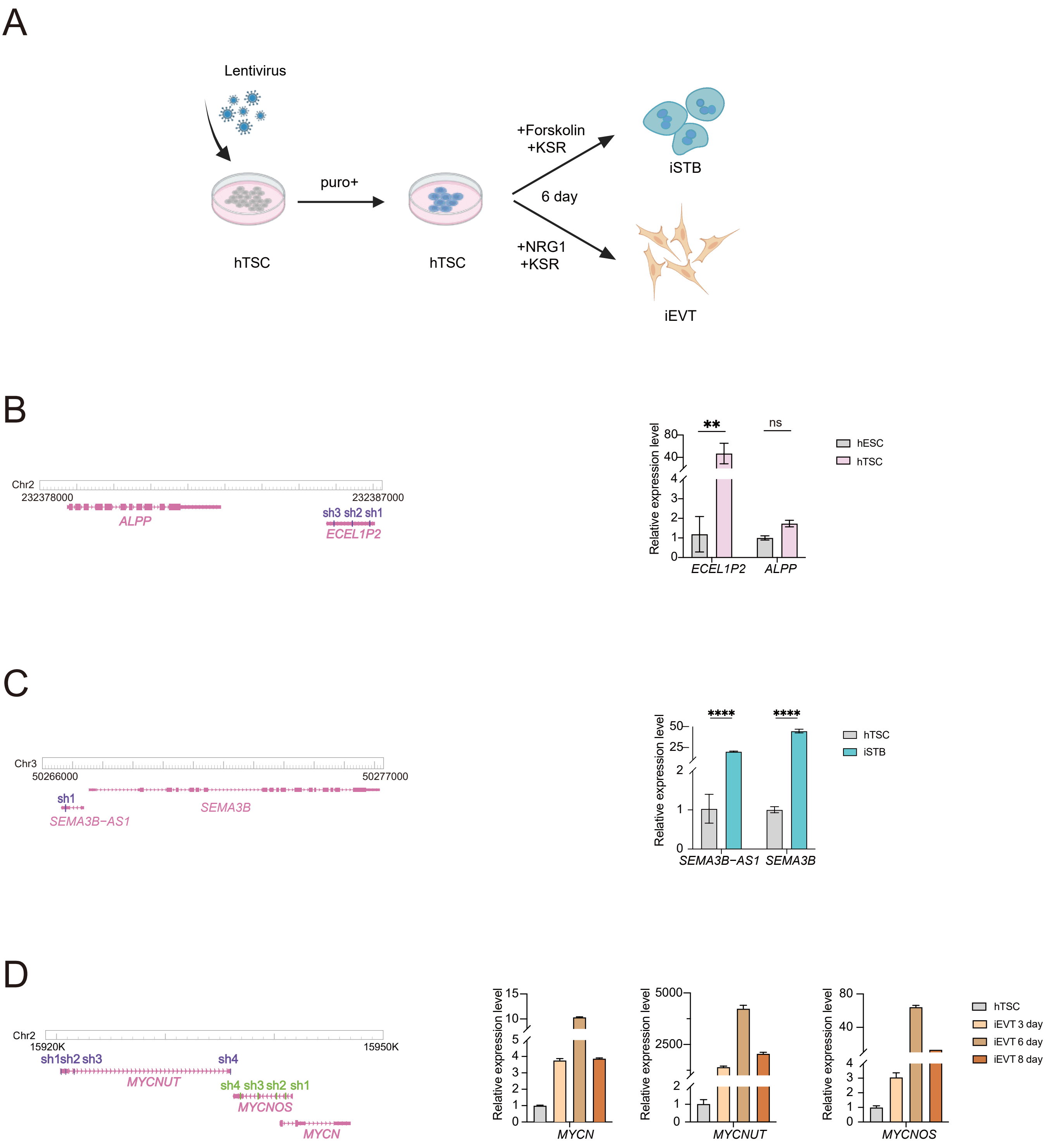

Supplement: lnag010_Supplementary_Data [file lnag010_supplementary_data.zip › 24-Mar-2026_013343_Supplementary_Material.zip!/Extended_Data_Figure 3_upload.pdf]
